# Comparison between curettage adenoidectomy and endoscopic-assisted microdebrider adenoidectomy in terms of Eustachian tube dysfunction^☆^

**DOI:** 10.1016/j.bjorl.2018.08.004

**Published:** 2018-09-25

**Authors:** Mahmut Huntürk Atilla, Selda Kargın Kaytez, Gülin Gökçen Kesici, Sibel Baştimur, Sebahattin Tuncer

**Affiliations:** Yıldırım Beyazıt University, Yenimahalle Training and Research Hospital, Ankara, Turkey

**Keywords:** Curettage adenoidectomy, Microdebrider adenoidectomy, Eustachian tube dysfunction, Adenoidectomia por curetagem, Adenoidectomia com microdebridador, Disfunção tubária

## Abstract

**Introduction:**

Adenoidectomy can be performed with many ways, including curettage and microdebrider endoscopic-assisted adenoidectomy. Those two techniques have advantages and disadvantages.

**Objective:**

The objective of this study is to research the effects of curettage adenoidectomy and endoscopic-assisted microdebrider adenoidectomy on the tympanum pressures in pediatric patients with adenoid hypertrophy without otitis media with effusion.

**Methods:**

This prospective descriptive study was performed with 65 patients who had a normal tympanic membrane and normal tympanogram and then underwent adenoidectomy or adenotonsillectomy for adenoid and tonsil hypertrophy. The subjects were randomly divided into two groups: curettage adenoidectomy group and endoscopic microdebrider-assisted adenoidectomy group. They underwent tympanometry, and the preoperative as well as 1st and 7th day postoperative values of the tympanum pressures were compared within and among the groups.

**Results:**

There were 32 patients in the curettage adenoidectomy group and 33 patients in the microdebrider adenoidectomy group. Statistically significant differences were observed in the median tympanum pressure on the preoperative and 1st and 7th postoperative days for both the left and right ears with curettage adenoidectomy (*p* < 0.001, *p *< 0.001). This difference occurred on the 1st postoperative day, and the value returned to normal on the 7th day. There was no significant difference in the median tympanum pressure on the preoperative and 1st and 7th postoperative days for both the left and right ears in the microdebrider adenoidectomy group (*p* = 0.376, *p* = 0.128).

**Conclusion:**

Postoperative Eustachian tube dysfunction is seen less often with the endoscopic-assisted microdebrider adenoidectomy technique than with the conventional adenoidectomy technique.

## Resumo

**Introdução:**

A adenoidectomia pode ser realizada de várias maneiras, inclusive por curetagem e por microdebridador, assistida por endoscopia. Essas duas técnicas têm algumas vantagens e desvantagens.

**Objetivo:**

O objetivo deste estudo foi investigar os efeitos da técnica de adenoidectomia por curetagem e da adenoidectomia por microdebridador assistida por endoscopia sobre a pressão timpânica em pacientes pediátricos com hipertrofia adenoideana sem otite média com efusão.

**Método:**

Estudo descritivo prospectivo feito com 65 pacientes que apresentavam membrana timpânica e timpanograma normais, que foram então submetidos à adenoidectomia ou adenotonsilectomia por hipertrofia adenoamigdaliana. Os pacientes foram divididos aleatoriamente em dois grupos: grupo adenoidectomia por curetagem e grupo adenoidectomia por microdebridador assistida por endoscópio. Todos os pacientes fizeram timpanometria e os valores das pressões do tímpano pré-operatórios e pós-operatórios no 1° e 7° dias foram comparados intragrupos e entre os grupos.

**Resultados:**

Foram incluídos 32 pacientes no grupo adenoidectomia por curetagem e 33 pacientes no grupo adenoidectomia com microdebridador. Diferenças estatisticamente significantes foram observadas na mediana da diferença entre a pressão timpânica no pré-operatório e no 1° e 7° dias de pós-operatório para ambas as orelhas, direita e esquerda, na adenoidectomia por curetagem (*p* < 0,001, *p* < 0,001). Essa diferença ocorreu no 1° dia do pós-operatório e o valor retornou ao normal no 7° dia. Não houve diferença significante na mediana entre pressão timpânica no pré-operatório e no 1° e 7° dias de pós-operatório para as orelhas direita e esquerda no grupo de adenoidectomia com microdebridador (*p* = 0,376, *p* = 0,128).

**Conclusão:**

A disfunção tubária no pós-operatório é observada menos frequentemente com a técnica de adenoidectomia por microdebridador assistida por endoscopia quando comparada com a técnica convencional.

## Introduction

Adenoidectomy is one of the most common surgical procedures performed in children.^1^ Adenoidectomy, a technique in which nasopharyngeal lymphoid tissue is removed, was first described as curettage (conventional) adenoidectomy in 1885.^2^ Standard adenoidectomy is performed utilizing an adenoid curette or adenotome.^3^ To observe the residual tissue at the end of operation, indirect visualization by a laryngeal or dental mirror or digital palpation is required. The main disadvantage of this method is that it is a relatively blind technique.^4^ When the procedure is not carried out by an experienced surgeon, residual adenoid tissue may be left behind at a high rate, especially in the choanal and tubal area: to remove this tissue may lacerate the Eustachian tube or pharyngeal muscle. While Saxby and Chappel^5^, ^6^ reported that the remaining residual tissue rate is 68% following curettage adenoidectomy, Ezzat et al.^7^ reported that it is 14.5%. Some part of the hyperplasic tissues may not be removed in the conventional curettage adenoidectomy technique if a bulky adenoid exists, particularly in intranasal extension and nasopharyngeal tissue above the adenoid tissue.^8^

With the increased use of intranasal endoscopy, endoscopes began to be utilized in adenoidectomy operations to ensure full removal of the adenoid bulk, achieve better hemostatic control with enhanced visualization and prevent possible damage.^9^ Endoscopes enable us to perform the adenoidectomy procedure under direct visualization. With this approach, we can prevent remaining residual tissue and tissue damage and enable us to achieve more precise hemorrhagic control.

There are several studies comparing curettage and endoscopy-assisted techniques in the literature.^6^, ^7^, ^10^, ^11^, ^12^, ^13^, ^14^, ^15^, ^16^ These studies generally include information on the residual tissue, operation duration, bleeding level, and cost comparison.

In this study, we aimed to compare curettage adenoidectomy and endoscopic-assisted adenoidectomy with microdebrider techniques in terms of the tympanum pressure changes in children without middle ear pathology after the operations.

## Methods

This study is a single-blind randomized controlled trial. At the beginning of the study, the sample size has been calculated 21 persons (for alpha level: 0.05 and power 90%). Sixty-five pediatric subjects who were examined in Training and Research Hospital, Otorhinolaryngology Policlinic between the dates of April 2016–September 2016 and who were diagnosed with adenoid vegetation, had a normal ear examination, underwent a tympanogram before operation and underwent adenoidectomy or adenotonsillectomy operations were enrolled in the study. All patients were diagnosed with adenoid vegetation with transnasal fiberoptic flexible endoscopy evaluation or lateral nasopharyngeal radiograph. Patients who underwent adenoidectomy or adenotonsillectomy were excluded from the study due to recurrent infection. All patients underwent otoscopic examination before the operation, and only children with a normal tympanic membrane and Jerger Classification Type A tympanogram were included in the study. The subjects were randomly divided into two groups; one group underwent curettage adenoidectomy (*n* = 32) and the other group underwent endoscopic-assisted adenoidectomy with a microdebrider (*n* = 33).

The study was performed in accordance with the principles of the Helsinki Declaration. Informed consent was obtained from the patients, and local ethics committee approval was obtained from Training and Research Hospital prior to the study (Protocol Code: 2016/27).

### Surgical techniques

Two experienced surgeons performed all operations. The patients were placed in the Rose position by placing a pillow under their shoulders after orotracheal intubation under general anesthesia. The patients underwent the operation with a cover to keep the mouth and nose open. A Crowe-Davis surgical retractor was used as a mouth gag. The adenoid tissue was excised using an adenoid curette with the curettage adenoidectomy technique. Curettage process was performed several times. Digital palpation was employed and the presence of residual adenoid was checked by using a laryngeal mirror. Having ascertained total removal, lavage was performed with normal saline using a 10 mm injector through the nose. Gauze pads moistened with saline, were placed on the nasopharynx from the mouth, and compression was applied for a few minutes. A tonsillectomy procedure was carried out following the adenoidectomy for patients who required tonsillectomy.

In endoscopic adenoidectomy with a microdebrider, the nasal cavity and nasopharynx were evaluated using a 0, 2.7 mm rigid fiber-optic endoscope with a video attachment. The shaver was the XPS Xomed Powered System with a 15° angled 2.9 mm Tricutblade and straight-through suction irrigation. The microdebrider was operated at 3000 rpm in the oscillating mode with auto irrigation. Under the transnasal endoscopic view, the microdebrider cannula was brought to the nasopharynx from the mouth. Adenoid tissue excision was performed starting from the choanal area through the rear wall of the nasopharynx. The torus tubarius was recognized and protected. Afterwards, gauze pads soaked with normal saline were placed on the nasopharynx from the mouth, and compression was applied for a few minutes. In the event of continued bleeding, coblator cautery was used. The tonsillectomy process was employed with the dissection method for patients who required tonsillectomy following the adenoidectomy procedure.

### Tympanometry

The middle ear pressure levels of patients were measured preoperatively and on postoperative days 1 and 7 by an audiologist using tympanometry (Interacoustics AZ-26 impedance audiometer, Interacoustics A/S, Assens, Denmark). The equipment used a probe tone frequency of 226 Hz and a positive and negative pressure sweep between +200 and −400 daPa. Both ears were examined before the test, and ear cerumen was removed from the external ear canal. Preoperative tympanometry was performed on the day before the surgery. Tympanometry was repeated on the first and seventh days following surgery. Patients with Type A tympanogram as per Jerger Classification before surgery were included in the study. Tympanogram tests with −100 or less middle ear pressure (Type C) were accepted as pathologic and considered to have Eustachian tube dysfunction.

In total, 130 ears from 65 patients were evaluated, and the right and left ears of the patients were evaluated separately.

### Statistical analyses

Data analysis was done using SPSS (Statistical Package for Social Sciences) for Windows 15.0 (SPSS Inc., Chicago, Illinois, USA) program. Fisher's exact test was used to compare the categorical variables. Student's *t* test was used to compare the age variables. The middle ear pressure level variables were not normally distributed according to the Kolmogorov–Smirnov normality test. Therefore, nonparametric tests were used to compare the middle ear pressure values. The Friedman test was used to separately compare the preoperative and postoperative day 1 and 7 tympanometric pressures in each group. The Wilcoxon Sign Test was performed to determine the difference between the tympanometric pressure measurement days (pre-, post1-, and post7-) with a Bonferroni correction. For comparison of the two groups in terms of the middle ear pressure level, the Mann–Whitney *U* test was used. Continuous variables are presented as the mean, standard deviation and minimum–maximum values. A *p*-value <0.05 was considered statistically significant.

## Results

There were 32 patients in the curettage adenoidectomy group and 33 patients in the microdebrider adenoidectomy group. There was no significant difference in the number of patients and male/female ratio in both groups (*p* = 0.708). No age difference was observed in terms of the groups (*p* = 0.357). No significant difference was observed in the groups in terms of the tonsillectomy operation ratio (*p* = 0.789) (Table 1).

**Table 1:** Baseline data.

	Curettage adenoidectomy	Microdebrider-assisted adenoidectomy	*p* value
Number of patients (*n*)	32	33	0.708^a^
Gender (female/male)	17/15	17/16	0.708^a^
Age month (mean ± SD)	90.43 ± 35.92	83.15 ± 26.78	0.357^b^
Tonsillectomy number (*n*)	9	11	0.789^a^

a Fisher's exact test.

b Student *t* test.

Cases for which the middle ear pressures was <100 daPa (Type C tympanogram) were considered pathologic and identified as Eustachian tube dysfunction. In the curettage adenoidectomy group, pathological decreases in the middle ear pressures of at least one ear were determined in 26 (81.2%) patients on postoperative day 1. Bilateral Eustachian tube dysfunction was detected in 19 (59.3%) patients, and unilateral Eustachian tube dysfunction was detected in 7 (21.8%) patients on postoperative day 1. Type B tympanograms were seen in the ears of two patients. Complaints of otalgia and/or aural fullness were noted by 21 (65.6%) patients on postoperative day 1. On postoperative day 7, bilateral Eustachian tube dysfunction in two patients and unilateral Eustachian tube dysfunction in 1 patient continued. A complaint of otalgia and/or aural fullness was defined by 2 (6.2%) patients on postoperative day 7.

In the microdebrider adenoidectomy group, pathological decreases in the middle ear pressures of at least one ear were determined in 8 (24.2%) patients on postoperative day 1. Bilateral Eustachian tube dysfunction was detected in 1 (3.03%) patient and unilateral Eustachian tube dysfunction was detected in 7 (21.2%) patients on postoperative day 1. Type B tympanogram was not seen in any ear. Complaint of otalgia and/or aural fullness was not voiced by any patient. On postoperative day 7, Eustachian tube dysfunction was not seen in any ear.

In the curettage adenoidectomy group, a significant difference was observed in the median middle ear pressures for both the right and left ears in the preoperative period and on postoperative days 1 and 7 (*p *< 0.001, *p *< 0.001). This difference occurred on postoperative day 1 and then disappeared by postoperative day 7 (Table 2, Table 3). In the microdebrider adenoidectomy group, there was no statistically significant difference in the median middle ear pressures for both the right and left ears in the preoperative period and on postoperative days 1 and 7 (*p* = 0.376, *p* = 0.128) (Table 2, Table 3).

**Table 2:** Comparison of curettage adenoidectomy with microdebrider-assisted adenoidectomy methods in terms of the median middle ear pressure for the right ear.

	Curettage adenoidectomy Middle ear pressure (daPa)		Microdebrider-assisted adenoidectomy Middle ear pressure (daPa)		*p*^a^
	Mean ± SD	Min–max	Mean ± SD	Min–max	
Pre-operation	−36.50 ± 27.06	−93.00 to 32.00	−33.18 ± 25.32	−83.00 to 15.00	0.520
Day 1 post-op	−161.90 ± 86.98	−351.0 to −7.00	−72.24 ± 93.46	−350.0 to 0.00	<0.001
Day 7 post-op	−37.87 ± 39.35	−178.0 to 12.00	−27.51 ± 25.79	−87.00 to 15.00	0.338
*p*^b^	<0.001		0.125		

a Mann–Whitney *U* test.

b Friedman test.

**Table 3:** Comparison of curettage adenoidectomy with microdebrider-assisted adenoidectomy methods in terms of the median middle ear pressure for the left ear.

	Curettage adenoidectomy Middle ear pressure (daPa)		Microdebrider-assisted adenoidectomy Middle ear pressure (daPa)		*p*^a^
	Mean ± SD	Min–max	Mean ± SD	Min–max	
Pre-operation	−40.09 ± 31.94	−98.00 to 36	−30.93 ± 24.15	−85.00 to 9.00	0.174
Day 1 post-op	−178.87 ± 95.56	−392.00 to −20	−75.06 ± 91.77	−371.00 to −2.00	<0.001
Day 7 post-op	−41.28 ± 51.80	−232.00 to 4	−27.15 ± 24.03	−98.00 to 9.00	0.446
*p*^b^	<0.001		0.376		

a Mann–Whitney *U* test.

b Friedman test.

When the curettage adenoidectomy and microdebrider adenoidectomy groups were compared in terms of the preoperative and postoperative day 1 and 7 findings, no difference was observed on the preoperative and postoperative day 7 for the left and right ears, while a difference was observed on day 1 (Table 2, Table 3).

## Discussion

Eustachian tube dysfunction is an early complication that develops after adenoidectomy and causes otalgia and aural fullness. However, there are no data regarding its mechanism, prevalence, predisposing factors or whether it develops according to the surgery type. In this study, tympanometry, which is an objective test, and middle ear pressure levels were used to compare the curettage adenoidectomy and microdebrider adenoidectomy methods in terms of Eustachian tube dysfunction. Significant impairment was observed in the middle ear pressures on postoperative day 1 in the curettage adenoidectomy group, and the impairment returned to normal on postoperative day 7. Complaints of otalgia and aural fullness were seen with impairment of middle ear pressures in the curettage adenoidectomy group. However, in the microdebrider adenoidectomy group, no postoperative change was observed in the middle ear pressures. Also complaints of otalgia and aural fullness were not seen in the microdebrider adenoidectomy group. While no difference was seen between the preoperative and postoperative day 7 results in the groups, there was a statistically significant difference observed on postoperative day 1 in the curettage adenoidectomy group due to impairment in the middle ear pressures.

Two previous studies have demonstrated the relationship between tonsillectomy operation and Eustachian tube dysfunction. Hone et al. observed a C tympanogram in 39% of the patients on postoperative day 1 following tonsillectomy.^17^ Holt et al. observed significant tympanogram abnormalities on postoperative day 1 according to the control group in their study wherein they evaluated the middle ear pressures of 22 subjects following tonsillectomy using tympanometry.^18^ Eustachian tube dysfunction may be related to local lymphoid drainage impairment following surgery or to impaired coordination between the nasopharyngeal and tubal muscles. In these two studies, patients underwent tonsillectomy only. In our study, the effect of tonsillectomy on Eustachian tube dysfunction was likely balanced in both groups because there was no difference in the tonsillectomy rate in the two groups.

The tympanometric results in the study show that Eustachian tube dysfunction complication is seen more frequently with the curettage adenoidectomy technique, while it is seen less frequently with the microdebrider adenoidectomy technique. Accordingly, the latter technique is a safe technique in terms of Eustachian tube dysfunction and ear complaints are not seen.

The Eustachian tube dysfunction that developed following curettage adenoidectomy was temporary, and it was mostly improved on postoperative day 7. The edema of the surgical site, especially the area around Eustachian tube, may lead to Eustachian tube dysfunction. In the curettage method, the surgery is performed blindly. Therefore, we think that applying digital palpation to identify the existence of residual tissue increases the risk of damage to the Eustachian tube and leads to more edema formation at the surgical site. In the literature, significant residual adenoid residues were identified after curettage adenoidectomy, especially in the torus tubarius and nasopharynx. Elnashar et al. identified adenoid residuals in 95.45% of the cases following curettage adenoidectomy.^19^ Abdel-Aziz identified residual adenoid tissue in 20.5% of the cases.^20^ Residual adenoid tissue, particularly around the torus tubarius, causes Eustachian tube dysfunction. Additionally, endoscopy is performed under visual control and we, therefore, think that leaving the surgical site cleaner and more efficiently removing blood clots will also be helpful for the microdebrider adenoidectomy technique.

Unlu et al. determined a pathologic decrease in the rate (75%) of least one middle ear pressure on the first postoperative day in pediatric patients who underwent adenoidectomy or adenotonsillectomy with the curettage method. This decrease was 15.6% on the third postoperative day, a change that was temporary.^21^ These findings are similar to the 81.2% prevalence rate that we determined on postoperative day 1 with the curettage adenoidectomy technique in our study. In our study, a significant change in the middle ear pressure was observed in children who underwent endoscope-assisted adenoidectomy with a microdebrider or adenotonsillectomy. We observed a pathologic decrease in the rate of 25% in the middle ear pressure for at least one ear on postoperative day 1 in this group. However, unlike in our study, Choi et al.^22^ determined the Type C tympanogram was 74% in a tympanometric examination that was performed on first day following operation in 25 children who underwent adenoidectomy with microdebrider or adenotonsillectomy. This result may be related to the surgical technique, and the biggest disadvantage of this study is the lack of a control group. More studies are needed in this field. In previous studies,^21^, ^22^ the tympanogram results of patients who only underwent curettage or endoscopy-assisted microdebrider methods were reported; however, in this study, we compared the results of pediatric subjects for the first time using both surgical methods.

## Conclusion

Temporary and short duration postoperative Eustachian tube dysfunction is seen less often with the endoscopy microdebrider-assisted adenoidectomy technique than with the conventional curettage adenoidectomy technique. We postulate the microdebrider procedure is better than the curettage adenoidectomy method in terms of prevention of Eustachian tube dysfunction in the first day after operation.

## Conflicts of interest

The authors declare no conflicts of interest.

## References

[1] Hall M.J., Lawrence L. (1998). Ambulatory surgery in the United States, 1996. Adv Data.

[2] Thornval A. (1969). Wilhelm Meyer and the adenoids. Arch Otolaryngol.

[3] Kornblut A.D. (1987). A traditional approach to surgery of the tonsils and adenoids. Otolaryngol Clin North Am.

[4] Pearl A.J., Manoukian J.J. (1994). Adenoidectomy: indirect visualization of choanal adenoids. J Otolaryngol.

[5] Saxby A.J., Chappel C.A. (2009). Residual adenoid tissue post-curettage: role of nasopharyngoscopy in adenoidectomy. ANZ J Surg.

[6] Cannon C.R., Replogle W.H., Schenk M.P. (1999). Endoscopic-assisted adenoidectomy. Otolaryngol Head Neck Surg.

[7] Ezzat W.F. (2010). Role of endoscopic nasal examination in reduction of nasopharyngeal adenoid recurrence rates. Int J Pediatr Otorhinolaryngol.

[8] Buchinsky F.J., Lowry M.A., Isaacson G. (2000). Do adenoids regrow after excision?. Otolaryngol Head Neck Surg.

[9] Becker S.P., Roberts N., Coglianese D. (1992). Endoscopic adenoidectomy for relief of serous otitis media. Laryngoscope.

[10] Koltai P.J., Kalathia A.S., Stanislaw P., Heras H.A. (1997). Scholarly articles for power assisted adenoidectomy. Arch Otolaryngol Head Neck Surg.

[11] Hartley B.E., Papsin B.C., Albert D.M. (1998). Suction diathermy adenoidectomy. Clin Otolaryngol Allied Sci.

[12] Yanagisawa E., Weaver E.M. (1997). Endoscopic adenoidectomy with the microdebrider. Ear Nose Throat J.

[13] Timms M.S., Ghosh S., Roper A. (2005). Adenoidectomy with the coblator: a logical extension of radiofrequency tonsillectomy. J Laryngol Otol.

[14] Owens D., Jaramillo M., Saunders M. (2005). Suction diathermy adenoid ablation. J Laryngol Otol.

[15] Havas T., Lowinger D. (2002). Obstructive adenoid tissue: an indication for powered-shaver adenoidectomy. Arch Otolaryngol Head Neck Surg.

[16] Viorel Z. (2011). Conventional curettage adenoidectomy versus endoscopic assisted adenoidectomy. J Clin Med.

[17] Hone S.W., Moodley S., Donnelly M.J., Fenton J.E., Gormley P.K., Walsh M. (1997). The effect of tonsillectomy on Eustachian tube function. Clin Otolaryngol Allied Sci.

[18] Holt G.R., Watkins T.M., Yoder M.G., Garcia A. (1981). The effect of tonsillectomy on impedance audiometry. Otolaryngol Head Neck Surg.

[19] Elnashar I., El-Anwar M.W., Basha W.M., AlShawadfy M. (2014). Objective assessment of endoscopy assisted adenoidectomy. Int J Pediatr Otorhinolaryngol.

[20] Abdel-Aziz M. (2012). Endoscopic nasopharyngeal exploration at the end of conventional curettage adenoidectomy. Eur Arch Otorhinolaryngol.

[21] Unlu I., Unlu E.N., Kesici G.G., Guclu E., Yaman H., Ilhan E. (2015). Evaluation of middle ear pressure in the early period after adenoidectomy in children with adenoid hypertrophy without otitis media with effusion. Am J Otolaryngol.

[22] Choi J.H., Yoon H.C., Kim T.M., Choi J., Park I.H., Kim T.H. (2015). The immediate effect of adenotonsillectomy on Eustachian tube function in children. Int J Pediatr Otorhinolaryngol.

